# Prediction of spontaneous closure of traumatic macular hole with spectral domain optical coherence tomography

**DOI:** 10.1038/srep12343

**Published:** 2015-07-21

**Authors:** Haoyu Chen, Weiqi Chen, Kangken Zheng, Kun Peng, Honghe Xia, Lei Zhu

**Affiliations:** 1 Joint Shantou International Eye Center, Shantou University and the Chinese University of Hong Kong, Shantou, China

## Abstract

It has been known that some traumatic macular holes can close spontaneously. However, knowledge about the types of macular hole that can close spontaneously is limited. In this retrospective study, we investigated patients with traumatic macular hole who were followed-up for at least 6 months without any surgical intervention. Clinical data and spectral domain optical coherence tomography (SD-OCT) images were compared between groups with and without macular hole closure. Overall, 27 eyes were included. Spontaneous closure of macular hole was observed in 10 (37.0%) eyes. The holes with spontaneous closure had smaller minimum diameter (244.9 ± 114.4 vs. 523.9 ± 320.0 μm, p = 0.007) and less intraretinal cysts (10% vs. 76.5%, p = 0.001) compared to the holes that did not close spontaneously. The area under the curve of receiver operative characteristic was 0.812 and 0.832 for minimum diameter of macular hole and presence of intraretinal cysts respectively. Multivariate logistic regression showed that the presence of intraretinal cysts was an independent predictive factor for closure of macular holes. The group with spontaneous macular hole closure had a high chance of visual improvement. Our study suggests that the absence of intraretinal cysts on SD-OCT can predict spontaneous closure of traumatic macular hole.

Macular hole is a full-thickness break or defect in neurosensory retina at fovea which leads to severe visual impairment. It can be idiopathic or secondary to ocular trauma, retinal detachment and laser. Idiopathic macular hole is the most common type of macular hole. On the contrary, traumatic macular hole is relatively rare and less widely reported. Macular hole can close spontaneously or can be repaired by vitreoretinal surgery. It is also known that an idiopathic macular hole rarely closes spontaneously and therefore mostly requires vitrectomy. The proportion of spontaneous closure in idiopathic macular hole has been reported to be 2.7–6.2%^1^,^2^. However, a relatively higher proportion (10.7% to 44.4%) of traumatic macular holes close spontaneously^3^,^4^. The mechanism of macular hole closure either spontaneously or by vitrectomy is not understood very well. There are no reported predictive factors for closure of macular hole. Therefore, it is difficult to decide whether to operate on this subgroup of patients.

Spectral domain optical coherence tomography (OCT) provides *in vivo* high resolution cross-sectional images of the microstructure of retinal tissue. It has been used to characterize idiopathic macular hole in order to understand its morphology, staging and pathogenesis. The purpose of this study is to explore whether the morphological characteristics on spectral domain OCT can be used to predict spontaneous closure of traumatic macular holes.

## Results

**Figure 1:**
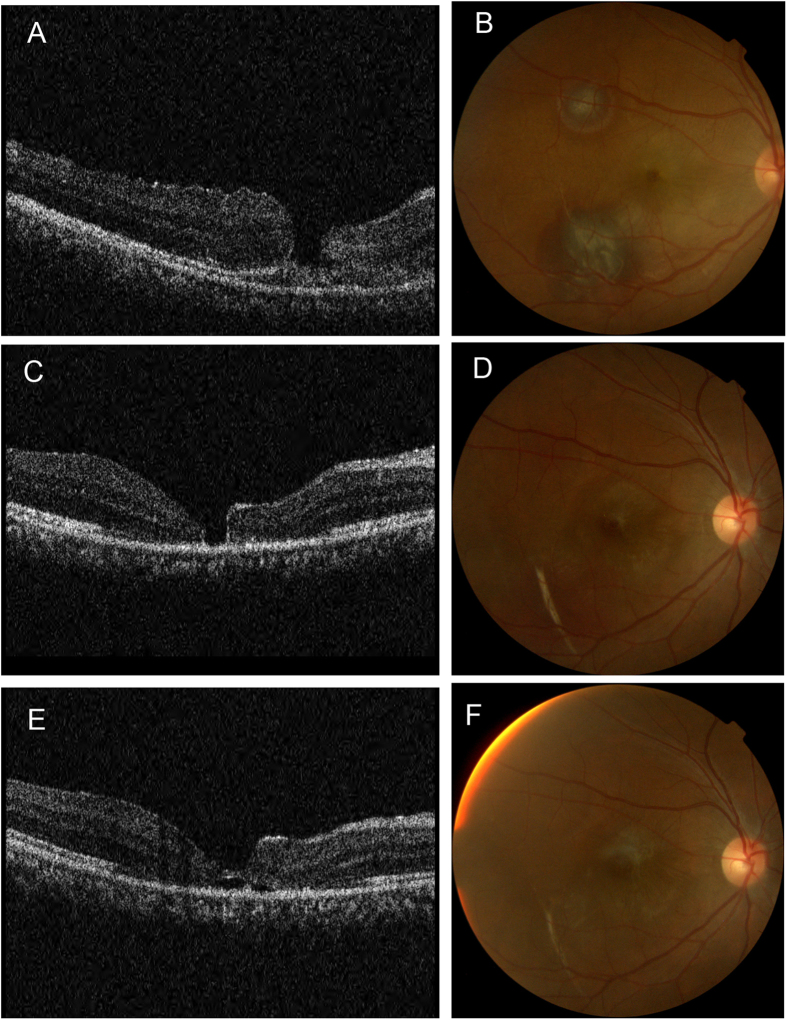
A case of traumatic macular hole with spontaneous closure. A 24 years old patient’s right eye was injured by a basketball. The best-corrected visual acuity was Finger Counting at 20 cm. Spectral domain OCT (**A**) and fundus photography (**B**) revealed traumatic macular hole and subretinal hemorrhage. The diameter of macular hole was 217 μm and there was no intraretinal cyst. Two months later (**C,D**), the hole became smaller and there was retinal atrophy around. Five months later (**E,F**), the hole closed spontaneously and the visual acuity improved to 20/400.

**Figure 2:**
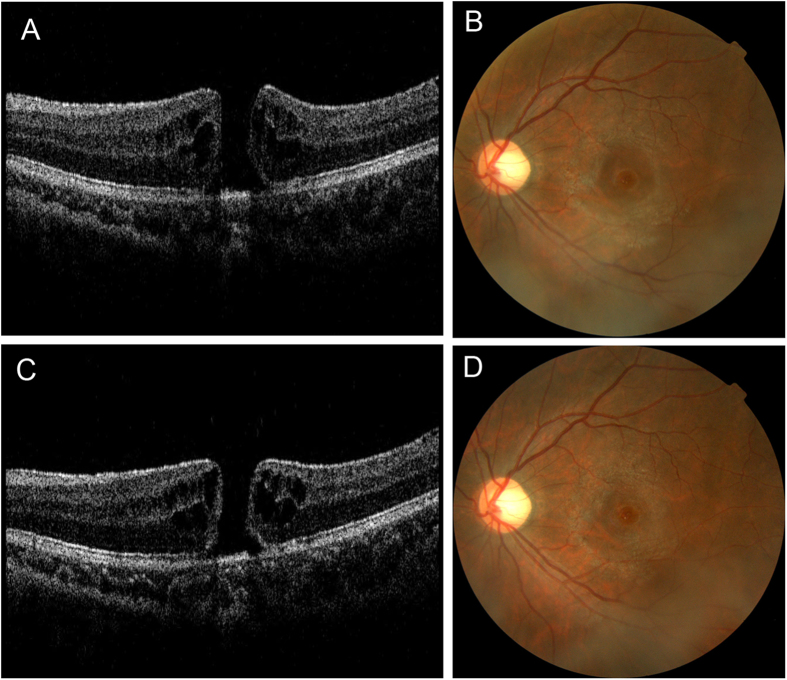
A case of traumatic macular hole without spontaneous closure. A 20 years old patient’s left eye was injury by fist. The best-corrected visual acuity was 20/120. Spectral domain OCT (**A**) and fundus photography (**B**) revealed traumatic macular hole. The diameter of macular hole was 354 μm and there was intraretinal cyst around the hole. After six months follow up (**C,D**), the size of macular hole did not change and the best-corrected visual acuity was 20/200.

Overall 27 eyes of 27 patients with macular holes were included in this study. There were 23 males and 4 females. The mean age of the patients was 26.2 ± 10.7 (range 12–48) years. The mean time from trauma to OCT scan was 5.3 days (range 1–15 days). The mean follow-up time was 270.6 (range 180–1124) days. During the study period, 10 (37.0%) holes closed spontaneously (Figs 1 and 2).

**Figure 3:**
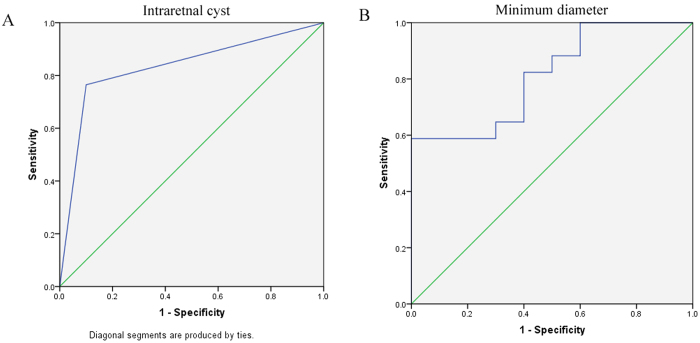
The Receiver Operating Characteristic (ROC) curves for minimum diameter (**A**) and intraretinal cyst (**B**) predicting spontaneous closure of traumatic macular hole.

The minimum diameter of traumatic macular holes with spontaneous closure (244.9 ± 114.4 μm) was smaller compared to those that did not close spontaneously (523.9 ± 320.0 um) (p = 0.007, Mann-Whitney test). The incidence of intraretinal cysts around macular hole was less in macular holes which closed spontaneously (1/10, 10.0%) compared to those that did not (13/17, 76.5%) (p = 0.001, Fisher’s exact test). No statistically significant difference was found in age, gender, time from trauma to OCT scan, base diameter, retinal thickness of any ETDRS region, accompanied retinal detachment, retinal atrophy, presence of vitreous traction or epiretinal membrane between both groups (Table 1). Multivariate logistic regression found that presence of intraretinal cysts was the only independent factor predicting spontaneous closure of macular hole (p = 0.005). The area under the receiver operating characteristic curve was 0.832 (95% confidence interval 0.668–0.997) and 0.812 (95% confidence interval 0.649–0.974) respectively, for the presence of intraretinal cysts and minimum diameter of macular hole (Fig. 3).

The BCVA improved from 1.36 ± 0.74 LogMAR at baseline to 1.01 ± 0.60 LogMAR at the last follow-up. The improvement of BCVA was greater in patients with macular hole closure (0.55 ± 0.48 LogMAR) compared to those that did not close (0.24 ± 0.82 LogMAR). The proportion of improvement in BCVA was 2-fold in patients with macular hole closure (9/10, 90.0%) compared to the patients without closure of holes (8/17, 47.1%) (p = 0.074, Fishers’ exact test.).

## Discussion

In this study, we found that 37.0% of the traumatic macular holes closed spontaneously over 6 months of follow up. Macular holes that closed spontaneously had an overall small minimum diameter and few intraretinal cysts. Multiple logistic regression showed that absence of intraretinal cysts was the only independent predictive factor for spontaneous closure of traumatic macular hole. Presence or absence of intraretinal cysts also had larger area under the ROC curve than minimum diameter of macular hole. Spontaneous closure of traumatic macular hole was associated with a better visual outcome compared to the macular holes that did not close.

Spontaneous closure of traumatic macular hole has been reported earlier in case reports^5^,^6^,^7^. There are only a few case series, which studied the natural history of traumatic macular hole. In 2002, Yamashita *et al.* reported 8 out of 18 (44.4%) traumatic macular holes that closed spontaneously after a mean follow-up of 8.4 months^3^. Li *et al.* reported 3 out of 28 (10.7%) traumatic macular holes which closed spontaneously after a mean follow-up of 18 months^4^. Faghihi reported 6 cases with spontaneous closure of traumatic macular hole after 1- to 6-month follow-up^8^. The rate of spontaneous closure of traumatic macular hole in our cases (37.0%) is comparable to the previous reports.

Our study identified two factors associated with spontaneous closure of traumatic macular holes. The first factor is small minimum diameter of the macular hole at baseline. It has been suggested earlier that small macular holes may resolve without any intervention^9^,^10^,^11^,^12^. Small macular defect may allow easy migration of glial cells. The other associated factor was absence of intraretinal cysts. In the earlier published case reports and case series, absence of intraretinal cysts has not been reported as a factor for closure of macular hole. However, most instances where traumatic macular holes closed spontaneously were not accompanied by intraretinal cysts on OCT^11^,^12^,^13^,^14^,^15^,^16^,^17^,^18^,^19^,^20^,^21^,^22^,^23^, although there are also a few cases with spontaneous closure in the presence of intraretinal cysts^24^,^25^,^26^. The presence of intraretinal cysts may be an indicator of internal limiting membrane traction, which may activate Müller cells and cause accumulation of extracellular fluid in the retina. Besides these two factors, young age has been reported to be associated with spontaneous closure of macular hole^20^. However, there was also a report of a 55-year old patient with traumatic macular hole that closed spontaneously^8^. In this case-control study, we did not find significant difference in age between the two groups. Since most of the published information has been from case reports or case series, there is insufficient evidence to conclude that young age is associated with spontaneous closure of macular holes.

Since significant proportion of traumatic macular hole close spontaneously with improvement in vision, it has been recommended that traumatic macular hole should be followed up and observed for at least several months before surgery to avoid unnecessary risk of surgical complications^3^. On the other hand, it has been reported that for idiopathic macular holes early surgery can result in a better functional outcome^27^. In this study we did not compare the functional outcomes after spontaneously closure and early surgical intervention. Further studies are needed to answer this question.

We recognize the limitations of this study. Firstly, the time from trauma to OCT scan is not consistent among patients. Although, there was no statistically significant difference between both groups, it may still be a confounding factor because of the limited sample size. Secondly, this is a retrospective study and the follow-up schedule was not consistent. OCT scan was not performed on a regularl basis, therefore we could not investigate the changes in the macular hole size or number of cysts during follow-up. Further prospective studies with larger sample size and regular OCT scan are needed.

In summary, more than one third of traumatic macular holes closed spontaneously in our study. Spontaneous closure of macular holes was associated with smaller minimum diameter of macular holes and absence of intraretinal cysts. Absence of intraretinal cyst is more valuable in predicting the outcome of traumatic macular hole.

## Methods

This retrospective study was approved by the Institutional Review Board of Joint Shantou International Eye Center (JSIEC), Shantou University and the Chinese University of Hong Kong. Informed consent was waived because of the retrospective nature of this study. The medical records database of JSIEC from July 2008, when we started using spectral domain OCT, to September 2014 was searched. The records of the consecutive patients who had undergone spectral domain OCT examination after closed globe injury were reviewed. The inclusion criteria were: (1) history of blunt ocular trauma; (2) traumatic macular hole confirmed by spectral domain OCT; (3) no vitreoretinal surgery performed within 6 months after diagnosis of macular hole; (4) follow up for 6 months.

The following examinations were performed: best-corrected visual acuity (BCVA), non-contact tonometry, slit-lamp biomicroscopy of anterior segment and retina, spectral domain OCT. Visual acuity was measured with the Chinese standard logarithmic visual chart and converted to Logarithm of minimal angle of resolution (LogMAR) units. A BCVA of finger counting (FC) was converted to 2.0 LogMAR and hand motion (HM) was converted to 2.3 LogMAR as suggested in the literature^28^.

Spectral domain OCT examination was performed using the Topcon 3D OCT-1000 (Topcon Corporation, Tokyo, Japan). Macula was scanned with the 6 *6 mm protocol, which consisted of 128 B scans. The axial and transverse resolution was 6-μm and 20-μm respectively. The minimum and base diameters of macular hole were measured using the caliper function of Topcon 3D OCT-1000 software. The retinal thickness at 9 ETDRS region was measured automatically. Accompanied conditions including choroidal rupture, retinal detachment, retinal atrophy, intraretinal cyst, vitreomacular traction, and epiretinal membrane were also recorded. Intraretinal cyst was defined as minimally reflective space within the neurosensory retina^29^. These parameters were obtained at baseline when traumatic macular hole was diagnosed. At the follow-up visits, status of macular hole closure was determined using OCT.

The main outcome measures were closure of macular hole at the last follow-up. Single variant analysis, such as Fisher exact test, Mann-Whitney test was used to compare the baseline characters, including age, gender, BCVA and spectral domain OCT data between the groups of macular hole with and without spontaneous closure. The parameters with p < 0.1 were included in binary multivariate logistic regression. P value < 0.05 was considered as statistical significance. Receiver operating characteristic (ROC) curves were derived for variables indicating good model discrimination, assessed by the area under the curve: values close to 1.00 represent good discrimination, whereas values close to 0.500 indicate that the model does not discriminate to any greater extent than that achieved by random allocation. The SPSS software (version 16.0, SPSS Inc. Chicago, IL) was used for statistical analysis.

## Additional Information

**How to cite this article**: Chen, H. *et al.* Prediction of spontaneous closure of traumatic macular hole with spectral domain optical coherence tomography. *Sci. Rep.* **5**, 12343; doi: 10.1038/srep12343 (2015).

## Figures and Tables

**Table 1:** Comparison of closed and unclosed traumatic macular hole on spectral domain optical coherence tomography.

	**closed**	**not closed**	**p**
Number	10	17	
Gender (Female: male)	2:8	2:15	0.613
Age (years)	25.9 ± 10.8	26.4 ± 10.9	0.764
Time from trauma to OCT scan	4.4 ± 2.4	5.7 ± 3.8	0.473
First BCVA (LogMAR)	1.48 ± 0.58	1.30 ± 0.83	0.264
Last BCVA (LogMAR)	0.93 ± 0.62	1.06 ± 0.61	0.749
BCVA change (LogMAR)	0.55 ± 0.48	0.24 ± 0.81	0.035
Minimum diameter (μm)	244.9 ± 114.4	523.9 ± 320.0	0.007*
Base diameter (μm)	1215.5 ± 1133.7	1408.9 ± 1057.9	0.386
Foveal thickness (μm)	319.8 ± 94.1	293.6 ± 149.9	0.664
Inner nasal thickness (μm)	358.3 ± 70.2	341.0 ± 113.1	0.402
Inner superior thickness (μm)	362.6 ± 45.4	314.6 ± 113.2	0.267
Inner temporal thickness (μm)	372.6 ± 73.2	325.4 ± 88.2	0.238
Inner inferior thickness (μm)	372.6 ± 68.1	319.0 ± 94.9	0.145
Outer nasal thickness (μm)	284.3 ± 55.0	307.3 ± 52.5	0.402
Outer superior thickness (μm)	277.6 ± 34.3	277.7 ± 53.8	0.764
Outer temporal thickness (μm)	289.3 ± 49.7	270.6 ± 51.0	0.197
Outer inferior thickness (μm)	288.1 ± 44.1	266.64 ± 48.6	0.616
Choroidal rupture (Yes: No)	2:8	4:13	1.000
Retinal detachment (Yes: No)	3:7	2:15	0.326
Retinal atrophy (Yes: No)	3:7	4:13	1.000
Intraretinal cyst (Yes: No)	1:9	13:4	0.001*
Vitreomacular traction (Yes: No)	0:10	1:16	1.000
Epiretinal membrane (Yes: No)	0:10	0:16	NA

BCVA: best-corrected visual acuity; NA: not applicable.
